# E-RCA – collation and feedback of HCAI information

**DOI:** 10.1186/1753-6561-5-S6-P236

**Published:** 2011-06-29

**Authors:** S Wallace, N Damani, R Rajendran

**Affiliations:** 1 Infection Control, Southern Health & Social Care Trust, Portadown, UK

## Introduction / objectives

Root Cause Analysis (RCA) is an analysis method which aims to identify the root causes of preventable Health care associated infections (HCAIs). It is based on the concept that problems are best solved by attempting to address and take corrective action by eliminating the *root causes* rather than merely addressing the immediately obvious issues.

The RCA tool developed by the UK Clean, Safe Care Team is useful helping to reduce MRSA (Methicillin resistant *S. aureus*) bacteraemias and *C. difficile* infections. We had been using RCA forms developed by the Clean, Safe Care Team until 2009 but felt that we needed to modify the content of the form to make it more effective and to develop a web based form to improve compliance with the RCA process.

## Methods

We felt there was a lack of clarity with some RCA questions based on the feedback from clinical staff who were involved in the RCA process. MRSA and *C.difficile* forms were extensively revised. Both forms were made available electronically. The form had a validation tool at every step making it impossible to submit an incomplete form and this has provided us with the relevant information which was necessary to analyse and make recommendations from the RCA process.

## Results

Since the introduction of E-RCA in March 2010 compliance has improved by 70%. The electronic form has allowed tracking of time delays within the system. The analysis of data was streamlined and simplified. Introduction of E-RCA forms has also increased ownership and accountability at both clinical and managerial level.

## Conclusion

It is recognized that for the RCA tool to be effective, corrective action must be taken in a *timely manner* and all the recommendations must be implemented to ‘close the loop’. As a result we have managed to reduce *C. difficile* and MRSA bacteraemia and meet Department of Health targets.

## Disclosure of interest

None declared.

